# The Effect of Low Temperatures on Environmental Radiation Damage in Living Systems: Does Hypothermia Show Promise for Space Travel?

**DOI:** 10.3390/ijms21176349

**Published:** 2020-09-01

**Authors:** Hisanori Fukunaga

**Affiliations:** 1 Patrick G Johnston Centre for Cancer Research, Queen’s University Belfast, 97 Lisburn Road, Belfast BT9 7AE, UK; hfukunaga01@qub.ac.uk; 2 Tohoku Medical Megabank Organization, Tohoku University, 2-1 Seiryo-machi, Aoba-ku, Sendai 980-8573, Japan

**Keywords:** environmental radiation, hibernation, HIF-1, hypothermia, hypoxia, radioresistance, ROS, space travel

## Abstract

Low-temperature treatments (i.e., hypothermia) may be one way of regulating environmental radiation damage in living systems. With this in mind, hibernation under hypothermic conditions has been proposed as a useful approach for long-term human space flight. However, the underlying mechanisms of hypothermia-induced radioresistance are as yet undetermined, and the conventional risk assessment of radiation exposure during hibernation remains insufficient for estimating the effects of chronic exposure to galactic cosmic rays (GCRs). To promote scientific discussions on the application of hibernation in space travel, this literature review provides an overview of the progress to date in the interdisciplinary research field of radiation biology and hypothermia and addresses possible issues related to hypothermic treatments as countermeasures against GCRs. At present, there are concerns about the potential effects of chronic radiation exposure on neurological disorders, carcinogenesis, ischemia heat failures, and infertility in astronauts; these require further study. These concerns may be resolved by comparing and integrating data gleaned from experimental and epidemiological studies.

## 1. Introduction

Since Wilhelm Röntgen’s discovery of X-rays in 1895 and Henri Becquerel’s discovery of radioactivity in 1896, countless studies have aimed to understand the characteristics of ionizing radiation (IR) and its effects on biological systems. Generally speaking, radiation is a double-edged sword: On the one hand, it has become an essential diagnostic and treatment tool in modern medicine; on the other hand, its carcinogenic properties are well-known. Radiation can induce a broad spectrum of DNA lesions, including single- and double-strand DNA breaks, damage to nucleotide bases, and cross-linking; further, exposure to radiation can seriously damage biological systems by triggering cell death or inducing mutations that lead to radiation-induced cancers [1]. The first radiation-related solid cancer was reported in 1902; it appeared in an ulcerated area of the skin. The first case of leukemia was documented in radiation workers in 1911 [2]. Almost immediately, animal model systems were applied to study the effects of radiation on what we now know as DNA. In 1927, Hermann Muller demonstrated that X-rays can cause mutations in fruit flies at a frequency with an approximately linear correlation with its dose [3]. According to monographs by the International Agency for Research on Cancer (IARC) [4], all types of IR have been classified as ‘Group 1’. This category is used to describe situations where there is sufficient evidence to expect carcinogenicity in humans. Thus, proper protection from IR is of importance for both human and non-human species.

Jacobi [5] was the first to develop the “effective dose” concept in 1975. Since then, it has since been used by the International Commission on Radiological Protection (ICRP) as a key measurement when assessing the risks of radiation exposure’s stochastic effects (e.g., hereditary effects and carcinogenesis) and dose exposure limits [6]. IR deposits energy directly into any matter being irradiated. The quantity used to express this energy is the absorbed dose; this is a quantified physical dose that is dependent on both the level of incidental radiation and the irradiated object’s absorption properties. The International System of Units (SI) has adopted the gray (Gy) as the unit for measuring absorbed doses. A ‘gray’ is defined as one joule of energy absorbed per kilogram of matter. As a physical quantity, an absorbed dose alone is not a satisfactory indicator of biological response, as responses may be driven by many accompanying factors. To accommodate the consideration of stochastic radiological risks, the ICRP and the International Commission on Radiation Units and Measurements devised the dose quantity’s equivalent dose and the effective dose. These are used to estimate the biological effectiveness of a given absorbed dose. The SI unit for an effective dose is the sievert (Sv), which currently represents, among the entire population, a 5.5% probability of developing cancer [6]. An ‘effective dose’ refers to the type of radiation and the characteristics of each organ or tissue that has been irradiated, since different organs have different levels of radiation sensitivity [7]. We know that the average annual effective dose from background radiation is around 3 mSv, while the typical effective doses of nuclear medical and radiology examinations are as follows: Standard radiographic examinations (approximately 0.01–10 mSv), most nuclear medicine procedures (0.3–20 mSv), computed tomographic examinations (2–20 mSv), and interventional radiological procedures (5–70 mSv) [8].

Low-temperature treatments (i.e., hypothermia) seem to be a promising means of regulating environmental radiation biological effects, although the underlying mechanisms of the approach remain unclear. It is well known that the hibernation of heterothermic, and the cooling of poikilothermic, animals in cold environments provides them with temporary protection against the acute effects of radiation [9]. Several studies over the past few decades have sought to explain the interactions of IR and temperature in animals, and there is evidence that hypothermia does provide at least some radioprotective effect. Some early studies showed that hypothermia in living organisms, such as fish (*Carassius carassius*) [10], frogs (*Rana pipiens*) [11], marmots (*Marmota monax*) [12], CF1 strain mice [13], T strain mice [14,15], rats [16], and ground squirrels (*Citellus tridecemlineatus*) [17,18], following total-body irradiation prolongs their survival. Furthermore, the radiosensitivity of certain organs in vivo and cells in vitro has been studied, such as the testes [19], spleen [20], ovaries [21], and the hemopoietic system [22]. All of these works were related to the acute effects of radiation.

In 1961, Bloch and colleagues were the first to prove that the irradiation of high-grade cerebral astrocytoma (glioblastoma multiforme) under conditions of mild whole-body hypothermia (rectal temperature maintained at 31–32 °C) led to an increase in radiosensitivity [23,24]. That same year, Joseph Weiss indicated the possibility that hypothermic treatments could increase a tumor’s radiosensitivity while decreasing the sensitivity of the normal tissues surrounding it [25]. The development of a system for evaluating localized hypothermia’s radioprotective effects is important for potential clinical applications [26]. A ground-breaking clinical study from 2017 showed the significant radioprotective effect associated with the use of localized hypothermia (15 °C) when accompanied by a single large dose of radiation aimed at mitigating a rectal obstruction and/or bleeding [27]. When used in tandem, whole-body and local hypothermic treatments have shown great promise for protecting normal tissue functions.

While it is known that hypothermia can have a protective effect against acute radiation injuries in living systems, it could also be useful for radiological protection in the future. This article aims to review recent progress in studies on the effect of hypothermia on environmental radiation damage in biomolecules, cells, and living systems. In the coming era of space travel, if hypothermia is shown to be capable of providing a radioprotective effect, hibernation could be an effective solution for those engaged in long-term missions. Here, from the point of view of radiological protection, we also discuss the possible impact of hypothermia on the future of space exploration.

## 2. Environmental Radiation Damage in Living Systems

The quality and quantity of DNA damage is assessed in terms of the radiation type and dose [28]. For example, high and low linear energy transfer (LET) radiation induce different spectra and qualities/complexity of DNA lesions, because of the differences in radiation track structures [29]. This also has an effect on the dose delivered to each cell by individual tracks at low doses, which is radiation quality dependent. Higher LET radiation delivers an increased dose per track. More specifically, in cases of low-dose exposure, such as environmental radiation, radiation’s energy deposition is localized along its track, which leads to a non-uniform distribution of exposed or unexposed cells in irradiated tissues [30,31]. For this reason, there are possible interactions between the irradiated and non-irradiated cells and the dynamics of those cells in the tissues that are involved in environmental radiation-induced biological responses at the whole-tissue level [32].

The “radiation-induced bystander effects (RIBEs)” refer to radiation-induced responses that are observed in cells that did not directly receive a radiation dose but did receive signals from nearby or neighboring irradiated cells. They behave as though they have been exposed, showing sister chromatid exchanges (SCEs) [33], chromosomal instability [34], micronuclei formations [35], gene mutations [36], and apoptosis [35,37]. In 1992, Nagasawa and Little first reported on RIBEs. They observed SCEs in ~30% of immortalized Chinese hamster ovary cells when only 1% of the population were calculated to be traversed through the nucleus by an a-particle following irradiation. Although most studies on bystander responses have reported cell damage in the non-irradiated cells, there are some reports on bystander-mediated adaptive responses [38]. Due to the complexity of these responses and the variety of positive and negative cellular endpoints, there is still controversy [39]. These responses are mediated either through gap junctions or via soluble factors released by irradiated cells.

In 1909, Köhler was the first to report on clinical observations of a tissue-sparing response during grid radiotherapy, wherein spatially fractionated radiation was delivered using a grid-like pattern of beams [40]. In 1995, a notable “tissue-sparing effect (TSE)” was reported in rat brain tissues during a study of microbeam radiotherapy (MRT) [41] performed at the National Synchrotron Light Source, at Brookhaven National Laboratory in Upton, New York. Since then, an MRT-related TSE has been confirmed in a variety of species and tissue types [40,41,42,43,44,45,46,47]. The TSE is the phenomenon by which normal tissues tolerate single exposures to narrow planes of synchrotron-generated X-rays up to several hundred Gy [48]. The TSE of spatial-fractionated radiation indicates significant implications for both clinical applications and the improvement of risk assessments for exposure to non-uniform radiation, such as environmental radiation.

Intercellular responses could be involved in non-targeted effects, including RIBEs and TSEs, in response to spatially fractionated radiation fields [49]. Furthermore, to achieve tissue homeostasis, cell competition is essential as a cell fitness-sensing mechanism. This is seen across an array of species, from insects to mammals. The process eliminates cells that, while viable, are less fit than their neighbors [50]. Damaged cells induced by spatially fractionated radiation are removed by the neighboring cells through cell competition, resulting in the prevention of a pathological state, such as carcinogenesis, at the tissue level [51]. After the complete clearance of radiation-induced damaged cells, tissue regeneration generally occurs for the purpose of maintaining normal tissue functions, namely homeostasis. Somatic stem cells migrate from the intact to the defective parts and regenerate the structure and function of the tissue via their proliferation and differentiation [52,53]. Such tissue homeostasis mechanisms could be involved in radiation-induced biological responses at the tissue level. However, there is little knowledge of this to date.

## 3. Mechanisms of the Effect of Hypothermia-Induced Radioprotection

Hypothermia is a condition that results from the drop in the core body temperature to a level at or below 35 °C. It develops when a body’s rate of heat production is exceeded by its rate of heat loss [54]. Physiological and environmental stresses that induce behavioral hypothermia include dehydration, hypercapnia, and anemia [55]. Given the marked effect that body temperature has on oxygen uptake in resting animals, hypothermia is beneficial in these conditions, as it reduces the demand for oxygen, decreases evaporative water loss (in amphibians), protects the brain’s metabolic status and function, and decreases xenobiotic compounds’ toxicity. In short, hypothermia elicits hypoxia.

It has long been known that the amount of available oxygen in tissues plays an important role in determining these tissues’ IR sensitivity; this is referred to as the “oxygen effect” [56,57]. In 1953, Louis Gray and his colleagues hypothesized that tumors are generally more anoxic than their surrounding normal tissues. As a result, since they are anoxic, tumors are found at a low position on the oxygen-radiosensitivity curve, when compared with normal well-vascularized tissues [56]. In fact, hypoxia can lead to an up to three-fold increase in radioresistance [58]. Furthermore, cellular antioxidants have shown a significant decrease of radiosensitivity, such as glutathione [59]. Thus, when discussing the effects of hypothermia on radiosensitivity, the degree of tissue oxygenation must be considered. In 1960, Joseph Weiss used aerobic cultures from HeLa cells, the first immortal human cell line, to demonstrate that there is no difference in radiosensitivity when cultures are irradiated at 1 and 37 °C [60]. He also revealed that no histological differences were detected between the spleens of mice irradiated under hypoxic hypothermic conditions [20] and those irradiated while hypoxic at a normal body temperature [61]. This indicates that hypothermia’s effect on irradiated mammalian cells and tissues is dependent on oxygen levels. From this, we can infer that the observed increase in radiosensitivity is mainly the result of concomitant hypoxia.

In clinical practice, hypoxia is a hallmark of solid tumors and a major obstacle to the effectiveness of radiotherapies, which kill cancer cells through the generation of reactive oxygen species (ROS) [62]. Several radiobiological studies have shown that hypoxic cells are resistant to ROS insults because of the shortage of ROS substrate oxygen. Further, and paradoxically, there is evidence that ROS are produced more in hypoxic than in normoxic cells and serve as signaling molecules that render cells adaptive to hypoxia. In 1995, Gregg Semenza’s research group discovered hypoxia-inducible factor (HIF-1) [63], which is involved in these hypoxic responses and regulates several genes in ROS homeostasis [64]. HIF-1 consists of an inducible alpha subunit (HIF-1α) and a constitutively expressed beta subunit (HIF-1β) [65]. Under normoxic conditions, the lysine and proline residues on HIF-1α’s oxygen-dependent degradation domain are hydroxylated, and the modified HIF-1α interacts with the Von Hippel-Lindau E3 ubiquitin ligase complex. This is followed by degradation via the ubiquitin proteasome pathway [66]. However, as previously described by Ridder and colleagues [67], hypoxia induces the accumulation of HIF-1α through the prevention of protein degradation, or by the upregulation of gene expression via ROS-mediated pathways. Then, as a result of increased HIF-1α, HIF-1 is activated and regulates more than 100 genes, conferring radioresistance by acting on multiple mechanisms at different levels. In addition, hypoxia- and radiation-induced ROS could trigger a feedback loop that favors the generation of antioxidants. In this way, the combination of hypoxia, ROS, and HIF-1 signaling demonstrates its important role in hypothermia-induced radioresistance for both tumors and normal tissues.

In non-uniform radiation fields, such as environmental radiation exposures, radiation-hit cells and non-hit cells co-exist. Regarding this, in vitro experimental configurations containing in-field and out-of-field cells have been established, showing that intercellular communications from cells in-field to cells out-of-field reduces the survival of out-of-field cells using a 50% in-field and 50% out-of-field (half-field) irradiation [68]. Under hypoxic conditions, while in-field responses were oxygen dependent, out-of-field effects were observed to be independent of oxygen, with similar or greater cell killing [69]. This highlighted the need for further understanding of intercellular signaling under hypoxic conditions.

There are other possible mechanisms for eliciting hypothermia-induced radioprotection. For example, when enzymes are exposed to IR at low temperatures, there is a progressive decrease in radiation sensitivity; namely, greater levels of enzymatic activity after the same dose of radiation delivered at a low temperature compared to room temperature [70]. Furthermore, cold-inducible RNA binding protein (CIRP), which responds to mild cold shock, assists cells in adapting to hypothermic conditions by stabilizing specific mRNAs and facilitating their translation. CIRP protects cells from ultraviolet radiation and hypoxia-induced senescence processes [71]; however, these radioprotective effects for maintaining cellular activity require further study and investigation.

## 4. Impairment of Radiation Damage Repair under Hypothermic Conditions

In 1966, Egami and Etoh applied lethal acute or fractionated exposures of radiation to fish (*Oryzias latipes*) at 23 and 11 °C [72]. At a water temperature of 23 °C, the delivery of a lethal dose of 40 Gy into two fractions, separated by a three-day interval, helped many fish to survive the 30-day experimental period; by contrast, at a lower water temperature, 11 °C, the overall response to the fractionated dose was the same as to the whole dose delivered at once. This indicates that, at low temperatures, the efforts to repair radiation damage injuries are ineffective; thus, a fractional dose does not help to reduce the radiation damage [73].

Poikilothermic animals are good experimental subjects for studies on the influence of low temperature on radiation sensitivity. According to a 1982 report by the United Nations Scientific Committee on the Effects of Atomic Radiation (UNSCEAR), tissue repair/regeneration and recovery from radiation injury in self-renewing tissues in fish are considerably inhibited when kept at suboptimal temperatures [74]. In cases of chronic radiation exposure, radiation damage accumulates without repair in fish in cold environments, such as Arctic winters. In contrast, those fish in warmer environments, which are continuously able to repair some of their radiation-induced damage, demonstrate lower levels of radiation damage from chronic exposure [9]. The impairment of radiation damage repair capacities may be involved in other living systems, including those of homoiothermic animals. In addition, other endpoints, specifically non-cancer outcomes, such as neurological disorders and ischemic heart disease, could benefit from further investigation.

The radiation-induced effects in genotoxicity under hypothermic conditions are yet to be determined. In recent years, Baird and colleagues showed that hypothermia (13 °C) postpones DNA damage repair in irradiated BJ-hTERT cells and protects against cell death [75]. By contrast, a series of studies investigating hypothermia-induced DNA damage in human peripheral blood lymphocytes [76,77,78,79,80] has suggested the possibility of DNA repair promotion that leads to a reduced transformation of DNA damage to chromosomal aberrations. Therefore, to our knowledge, hypothermia-induced modulations of DNA damage repair in vitro remain controversial.

## 5. Possible Issues Related to Human Space Exploration and the Induction of Hibernation

In 1961, Yuri Gagarin was the first human to journey to outer space. Since then, the technology associated with space travel and exploration has advanced significantly. As future missions explore realms beyond low-Earth orbit (LEO) and outside the protection zone of the Earth’s magnetic shielding, the nature of the radiation exposure that astronauts encounter will include higher levels of exposure [81]. While travelling outside LEO, every cell in an astronaut’s body will be traversed by proton or electron rays every few days, and by high atomic number and energy (HZE) ion-charged particles every few months [82]. Shielding may be able to reduce their exposure to galactic cosmic rays (GCRs), including HZE particles, but the technique is not likely to resolve the problem entirely [83]. From the viewpoint of radiation micro-dosimetry, for GCR exposures, the energy deposition of radiation is localized along its track. This results in a spatially fractionated distribution of exposed or unexposed cells in the irradiated tissue. For this reason, the detection of the temporospatial dose’s distribution could be of scientific importance, allowing for more accurate individual risk assessments of exposure associated with environmental radiation.

The concept of the effective dose does not yield an individual-specific dose but instead uses a reference person for a given exposure situation. Furthermore, effective doses do not take into account the genomic diversity of individuals’ radiosensitivity, so it is not an appropriate means for estimating individuals’ radiation-induced health risks among the general population. In fact, as explained in *ICRP Publication 103*, the effective dose is a risk-adjusted quantity for the control of exposure; it was never intended to serve as a measure of risk [6]. This dose is calculated using reference phantoms for the purpose of enabling the summation of doses from all radiation exposures for comparisons with limits, constraints, and reference levels (set at the same quantity), and for the optimization of protection. Implicit in its use is the central assumption of a linear non-threshold dose–response relationship between dose and risk; it is a reasonable assumption for protection purposes but has not been proven for low doses [84]. A single set of tissue-weighting factors is used in the calculation of the effective dose, despite previously recognized differences in the age and sex dependence of the relative contributions of cancer types to the overall detriment and, crucially, in the overall magnitude of cancer detriment.

Risk assessment and proper GCR protection are essential for humans’ long-term activities in space [85]. In the US, the National Aeronautics and Space Administration (NASA) bases its safety standards on the acute exposure levels recorded among Japanese atomic bomb survivors [86], but this ultimately seems to be an insufficient approach, as the survivors’ acute irradiation scenario differs from the chronic exposure to GCRs that astronauts on a two- or three-year mission to Mars would encounter [87]. In addition, physiological factors, such as age, sex, and DNA repair-deficiency, are clearly important in estimating the biological effects induced by exposure to radiation. In fact, NASA has developed a risk-based approach to radiation exposure limits that accounts for individual factors (e.g., age, gender, and smoking history) and uses them when assessing the uncertainties related to risk estimates [88]. However, many more epidemiological studies are still needed; the Million Person Study of Low-Dose Health Effects (MPS) [89], for example, can contribute to our understanding of the health effects of chronic exposure. The MPS’ large study size, approximately one million subjects, makes it capable of providing more precise estimates of lifetime risks of radiation and may indicate reasonable approaches for addressing specific issues of interest to not only NASA but also ICRP, UNSCEAR, and other organizations concerned with radiological protection.

The idea of hibernation has been proposed as a possible approach for use in human space travel. Recently, a procedure to induce a metabolic state known as “synthetic torpor” in non-hibernating mammals was successfully developed; this could be an efficient means of conserving resources, reducing the incidence of mental disorders related with long-term missions and mitigating or preventing radiation-induced acute effects [90]. However, GCR-induced long-term or chronic health risks for humans, such as cancer, heart failure, and dementia, during hibernation or synthetic torpor are still unclear. In addition, environmental radiation damage on reproductive potential remains to be determined. An epidemiological study from 2013 examined 83 healthcare workers who had been exposed to radiation at work. The study shed light on the effects of this exposure on spermatozoa, noting changes in their motility characteristics, global hypermethylation, increased incidences of morphological abnormalities in sperm, and sperm DNA fragmentation [91]. Such epidemiological investigation is of importance to provide new insights into environmental radiation risks on human fertility [92]. If a hypothermic state can induce an impairment of the radiation-induced damage repair response, hibernating astronauts might be more vulnerable to the chronic effects of radiation, compared to those allowed to travel under optimum-temperature sleeping conditions. At present, there is a lack of experimental studies on the combination of chronic radiation exposure and hypothermia. Taken together, current concerns about the possible effects of chronic radiation exposure on carcinogenesis, neurological disorders, ischemic heat failures, and infertility for astronauts require further elucidation that may be resolved by comparing and integrating observed data from epidemiological and experimental studies, as well as biophysical models and computational approaches.

## 6. Conclusions

**Figure 1:**
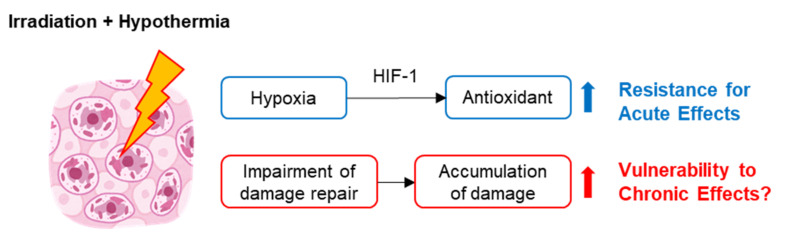
Overview of radiation-induced effects in cells in hypothermic conditions. Hypothermia treatments protect organisms from the effects of acute radiation via a hypoxic response. Additionally, it can induce the impairment of radiation damage repair responses.

This literature review offers a summary of the historical progress in the interdisciplinary research field of radiobiology and hypothermia. As shown in Figure 1, conventional hypothermia appears to be a double-edged sword: On the one hand, it protects organisms against the acute effects of radiation via a hypoxic response; on the other hand, it can lead to impaired radiation damage repair capabilities. Long-term or chronic radiation-induced health risks, including carcinogenesis, neurological disorders, ischemic heart disease, and sterility under hypothermic conditions, are still controversial; nevertheless, it is important for humans’ long-term activities in space.

To establish a personalized risk assessment for environmental radiation exposures, we should consider the tissue-level responses induced by heterogenous tempo-spatial radiation exposure as well as individual differences in radiation sensitivity. In addition, further investigations are required to provide novel insights into our understanding of the temperature regulation of environmental radiation damage in living systems, as this will be of increasing importance in the coming era of space exploration. Consequently, it will be of importance to monitor not only the total dose but also the tempo-spatial distribution as well as the temperature in the circumstances of the irradiation for risk assessment.

## Abbreviations

IR	Ionizing radiation
IARC	International Agency for Research on Cancer
UV	Ultraviolet radiation
ICRP	International Commission on Radiological Protection
SI	International System of Units
Gy	Gray
Sv	Sievert
LET	Linear energy transfer
RIBE	Radiation induced bystander effect
SCE	Sister chromatid exchanges
TSE	Tissue-sparing effect
MRT	Microbeam radiotherapy
ROS	Reactive oxygen species
HIF	Hypoxia inducible factor
CIRP	Cold-inducible RNA binding protein
UNSCEAR	United Nations Scientific Committee on the Effects of Atomic Radiation
LEO	Low-Earth orbit
HZE	High atomic number and energy
GCR	Galactic cosmic ray
NASA	National Aeronautics and Space Administration
MPS	Million Person Study of Low-Dose Health Effects
